# *Candida* Infection as an Early Sign of Subsequent Sjögren's Syndrome: A Population-Based Matched Cohort Study

**DOI:** 10.3389/fmed.2021.796324

**Published:** 2022-01-21

**Authors:** Chia-Lun Chen, Fang-Cherng Chang, Yao-Min Hung, Mei-Chia Chou, Hei-Tung Yip, Renin Chang, James Cheng-Chung Wei

**Affiliations:** ^1^Department of Emergency Medicine, Kaohsiung Veterans General Hospital, Kaohsiung, Taiwan; ^2^Department of Internal Medicine, Kaohsiung Municipal United Hospital, Kaohsiung, Taiwan; ^3^Shu-Zen Junior College of Medicine and Management, Kaohsiung, Taiwan; ^4^Institute of Medicine, Chung Shan Medical University, Taichung, Taiwan; ^5^Department of Recreation and Sports Management, Tajen University, Pingtung, Taiwan; ^6^Department of Physical Medicine and Rehabilitation, Kaohsiung Veterans General Hospital, Pingtung Branch, Pingtung, Taiwan; ^7^Department of Physical Therapy, Shu-Zen Junior College of Medicine and Management, Kaohsiung, Taiwan; ^8^Graduate Institute of Bioresources, National Pingtung University of Science and Technology, Pingtung, Taiwan; ^9^Management Office for Health Data, China Medical University Hospital, Taichung, Taiwan; ^10^College of Medicine, China Medical University, Taichung, Taiwan; ^11^Institute of Public Health, National Yang Ming Chiao Tung University, Hsinchu, Taiwan; ^12^Division of Allergy, Immunology and Rheumatology, Chung Shan Medical University Hospital, Taichung, Taiwan; ^13^Graduate Institute of Integrated Medicine, China Medical University, Taichung, Taiwan

**Keywords:** *Candida* infection, population-based cohort study, Sjögren's syndrome, NHIRD, EPI-epidemiology

## Abstract

**Background:**

*Candida* infection is prevalent in patients with Sjögren's syndrome (SjS), which usually takes years to reach diagnosis. Is the link a two-way street? The role of *Candida* infection before SjS has not been examined clearly. This study was conducted to provide epidemiological evidence regarding the relationship between the first acquisition of *Candida* infection and subsequent SjS.

**Methods:**

Totally, 23,494 individuals newly diagnosed with *Candida* infection were enrolled from 2000, to 2012. Controls (*N* = 93,976) were selected at a 1:4 ratio through propensity score matched (PSM) using the greedy algorithm. Exposure was defined according to the *International Classification of Diseases, Ninth Revision, Clinical Modification (ICD-9-CM)* codes.

**Main Outcomes and Measures:**

SjS was recorded in the Registry for Catastrophic Illness Patients Database (RCIPD). Cox proportional hazard model was used to analyze the association and sensitivity analyses for cross-validation.

**Results:**

Of 117,470 individuals (106,077 [89%] women), 23,494 individuals (20.0%) had *Candida* infection and 104 individuals (0.1%) developed SjS. The incidence of SjS was higher in the exposed group compared with the controls (1.92 vs. 0. 98 per 10,000 person-years) with adjusted hazard ratio (aHR) 1.90 (95% CI, 1.25–2.87). The aHRs in subgroups of aged 18–30 years, oral candidiasis and depression were 4.30 (95% CI, 1.60–11.55), 4.70 (4.70–13.93) and 6.34 (2.16–18.66). Sensitivity analyses yield consistent results.

**Conclusions:**

Residents in Taiwan with *Candida* infection have higher risk of SjS. For early diagnosis of SjS, clinicians are advised to take *Candida* infection into account in some situation.

## Key Messages

- Sjögren's syndrome (SjS) is a chronic, slow-progressing disorder, with no single diagnostic test could confirm the diagnosis.
- Some infections can trigger autoimmunity and the pathogenetic process of SjS.
- This study suggests that *Candida* infection is associated with a higher incidence of SjS, which may be considered for the early diagnosis of SjS.

## Introduction

Sjögren's syndrome (SjS) is a chronic, slow-progressing disorder with an extremely variable clinical manifestation that varies from a specific organ to a systemic autoimmune disease (1). The incidence of SjS varies widely from 3 to 11 cases per 100,000 individuals (2–5) and is highest in Asian women (6). However, the global incidence of SjS may have been underestimated, as some patients are asymptomatic or have mild symptoms and they may never get diagnosed. SjS adversely affects the quality of life of the patients; it also leads to a greater use of medical services by the patients, with an average total direct cost of US$20,000 per patient per annum in the United States (7). In spite of this, however, no test has yet been designed to definitively diagnose the SjS. Patience is the key when it comes to diagnose patients who initially report multiple instances of vague discomfort, which may delay the diagnosis of SjS by years. Many patients report numerous non-sicca features 20 years prior to the onset of typical dryness associated with the SjS (8). Hyposalivation is one of the most common symptoms of the SjS and *Candida* is the most prevalent yeast infection (9). Most of the previous studies focused on the prevalence and treatment of *Candida* infection after SjS; only a few studies examined the risk of SjS developing in patients who already have *Candida* infection. It has recently been shown that infections can trigger autoimmunity and begin the pathogenetic process of SjS (10). We hypothesized that *Candida* infection can be present before the occurrence of SjS or in the early state of undiagnosed SjS and is associated with the development of SjS. Herein, we investigate the association between SjS and *Candida* by using a population-based database with up to 14 years of follow-up and by analyzing the cumulative incidence, as well as by performing stratified analysis based on age, sex, and site of infection.

## Methods

### Data Source

The present study is a retrospective cohort study designed using the Longitudinal Health Insurance Database 2000 (LHID2000), a subset of Taiwan National Health Insurance Research Datasets (NHIRD). LHID2000 comprises data of 1,000,000 people randomly sampled from the year 2000 registry of all beneficiaries of the National Health Insurance. The NHIRD is a database of the medical claims (including demographics, diagnoses for various diseases, procedures, medication prescription, and in-patient and out-patient expenditure claims) of all National Health Insurance beneficiaries (11). No statistically significant difference was observed in the distribution of age and sex between the LHID2000 and original NHIRD databases (http://nhird.nhri.org.tw/date_cohort.htm). The health database has a characteristically large sample size of longitudinal nature and hence is suitable for non-experimental observational studies (12). Disease diagnoses were coded based on the 9th International Classification of Diseases (ICD-9). The Bureau of National Health Insurance regularly conducts quarterly expert-reviews on a random sample comprising the claims of out-patient and in-patient departments of all medical institutions of Taiwan. The Registry for Catastrophic Illness Patient Database (RCIPD) is a sub-dataset of the NHIRD, is linked with the NHIRD. Those who meet the criteria for catastrophic illnesses as defined by the Ministry of Health and Welfare, Government of Taiwan, can apply for a catastrophic illness certificate, and their information will be uploaded to the RCIPD. Patients should provide records of their diagnosis and the pathological or radiological report confirming the diagnosis, which are verified by an expert review committee. Individuals who qualify for the catastrophic illness-related category are not required to pay for their treatment.

The identification was scrambled to protect the privacy of the patients. This study was approved by the Institutional Review Board of China Medical University Hospital Research Ethics Committee [CMUH104-REC2-115(AR-4)].

### Study Participants

The participants were the patients newly diagnosed with *Candida* infection between 2000 and 2012, as identified from the LHID2000 based on ICD-9 codes (ICD-9 code 112). The participants should have at least three out-patient department visits or any hospitalization. The index date was defined as the date on which the *Candida* infection was first diagnosed; this date was then assigned to the matched non-*Candida* subjects with identical enrolment criteria. Patients with a history of *Candida* infection and those with SjS before the index date were excluded. Patients with incomplete information about their age and/or sex were also not considered. The control group comprised patients who had never been diagnosed with *Candida* between 1997 and 2012 from the same dataset. Propensity score matching (PSM) was performed for the pairs groups (control and study group) at a 4:1 ratio based on the individual age-, sex-, index years-, covariates-matching, including hypertension (ICD-9-CM codes 401-405), diabetes mellitus (DM, ICD-9-CM code 250), hyperlipidaemia (ICD-9-CM code 272), cerebral vascular accident (ICD-9-CM code 430–438), chronic kidney disease (CKD, ICD-9-CM code 585), chronic obstructive pulmonary diseases (COPD, ICD-9-CM codes 491, 492, 496), chronic liver diseases (CLD, ICD-9-CM code 571.x), depression (ICD-9-CM codes 296.2, 296.3, 298.0, 300.4, 311), allergic rhinitis (ICD-9-CM code 477.x), urticaria (ICD-9-CM code 708.x), systemic lupus erythematosus (SLE, ICD-9-CM code 710), and rheumatoid arthritis (RA, ICD-9-CM code 714.0). The data on these comorbidities were collected during the baseline period (i.e., within 2 years before the index date). Increasing the PSM ratio increases the statistical power, and such an effect is slightly advantageous when the ratio is >4 (13). To balance the baseline characteristics and relevant covariates between the two groups, greedy algorithm on the propensity score was applied (14). For each participant in the study group, the corresponding controls were selected based on the nearest propensity scores.

### Outcome and Relevant Variables

The main outcome is the diagnosis of new-onset SjS (ICD-9 code 710.2) extracted from LHID2000 and linked to RCIPD for confirmation during the follow-up period. The diagnosis was based on the classification criteria established by the European Community Study Group in 1993 (15). The ICD coding algorithm was used (16). All the participants were followed until the confirmed diagnosis of SjS, their death, or the end of 2013. The date of the determination of the first principal ICD-9 code of SjS during the follow-up period was defined as the endpoint of interest. To address potential measurable confounders, we included the following data into the regression model: sociodemographic factors (sex, age, and occupation) and baseline medical comorbidities (including hypertension, DM, hyperlipidaemia, CVA, CKD, COPD, CLD, depression, allergic rhinitis, urticaria, SLE, and RA).

### Statistical Analysis

The Chi-square (χ^2^) test was applied to process the demographic characteristic data, including the distributions of categorical sex, age, occupation, comorbidities between the *Candida* group and non-*Candida* group to check the category variables from the two groups. Person-years were calculated based on the amount of the follow-up time required for each individual, which was the period recorded from the index date to the end point of the study. The incidence density of SjS per 10,000 person-years was calculated for both groups. To investigate the independent association of *Candida* infection with SjS, a multivariable Cox proportional hazard regression analysis was conducted to estimate the hazard ratio (HR) with the 95% confidence interval (CI) after adjusting for full covariates. In this study, only the variables found to be statistically significant in the univariable analysis were further examined in the multivariable model (i.e., sex, age, occupation, hypertension, hyperlipidaemia, CVA, COPD, CLD, depression, allergic rhinitis, urticaria, SLE and RA). The Kaplan–Meier method was used to describe the cumulative incidence of SjS between the two groups. Differences in the incidence between the two groups were evaluated using the log-rank test. Sensitivity analysis I (to exclude the coincidence of SLE or RA developed from the index date to 2013, the end of the study) was conducted to re-evaluate the HR of SjS after exposure to *Candida* infection. Sensitivity analysis II was conducted using case-control analysis to identify patients with SjS and matched controls in the same database from 2000 to 2013. The multivariable conditional logistic regression model was used in sensitivity analysis II to examine the odds ratio (OR) of the previous exposure to *Candida* infection. Sub-group analysis was conducted based on the age, sex, and site of *Candida* infection. All the data and statistics were processed and analyzed using the SAS software Version 9.4 (SAS Institute, Inc., Cary, NC). All statistical tests were 2-sided, and *P* < 0.05 was considered statistically significant.

## Results

### Demographic Characteristics and Comorbidities

Of the 117,470 individuals (106,077 [89%] women) aged over 18 years, 23,494 (20.0%) had *Candida* infection (2,1981 women [90%]; mean [SD] age 37.8 [16.0] years) and 104 (0.1%) developed SjS; 93,976 individuals who had no *Candida* infection (84,096 women [89%]; mean [SD] age 37.0 [15.7] years) were matched by age, sex, and comorbidities (Table 1). Among the 23,494 participants with *Candida* infection, 18,960 (80.0%) were aged 50 years or younger. PSM resulted in 93,976 matched individuals in each group. The baseline characteristics were found to be well balanced between the groups after PSM. The group with *Candida* infection in this study, compared with that without *Candida* infection, had similar proportions of concomitant SLE (89 individuals [0.4%] vs. 313 individuals [0.3%]; SMD, 0.008) and RA (380 individuals [2%] vs. 1,299 individuals [1%]; SMD, 0.019).

**Table 1:** The demographic variables and comorbidities of patients with and without *Candida* infection.

	**Non-*****Candida*** **infection**		**Candida infection**		
	***N*** **= 93,976**		***N*** **= 23,494**		
**Variables**	***n***	**%**	***n***	**%**	**SMD**
Sex					0.009
Female	84,096	89%	21,981	90%	
Male	9,880	11%	2,537	11%	
Age, year					
18–30	40,144	43%	9,481	40%	0.048
31–40	22,760	24%	5,965	25%	0.027
41–50	13,015	14%	3,514	15%	0.032
>50	18,057	19%	4,534	19%	0.002
Mean, (SD)	37.0	(15.7)	37.8	(16.0)	0.050
Occupation					
Officers	50,642	54%	12,449	53%	0.018
Worker	24,334	26%	6,295	27%	0.020
Fisher	12	0.01%	4	0.02%	0.003
Farmer	10,743	11%	2,663	11%	0.003
Other	8,245	9%	2,083	9%	0.003
Comorbidities					
Hypertension	13,172	14%	3,281	14%	0.001
Diabetes	7,983	8%	2,050	9%	0.008
Hyperlipidemia	9,122	10%	2,388	10%	0.015
CVA	4,717	5%	1,238	5%	0.015
CKD	897	1%	251	1%	0.011
COPD	6,974	7%	1,781	8%	0.006
CLD	8,165	9%	2,093	9%	0.008
Depression	4,558	5%	1,173	5%	0.007
Allergic rhinitis	20,300	22%	4,923	21%	0.016
Urticaria	17,164	18%	4,212	18%	0.009
SLE	313	0.3%	89	0.4%	0.008
Rheumatic arthritis	1,299	1%	380	2%	0.019

*SMD, standardized mean difference; CVA, cerebrovascular accident; CKD, chronic kidney disease; COPD, chronic obstructive pulmonary disease; CLD, chronic liver diseases; SLE, systemic lupus erythematosus*.

### Comparison of SjS Incidence and HR Between *Candida* and Control Groups

**Figure 1:**
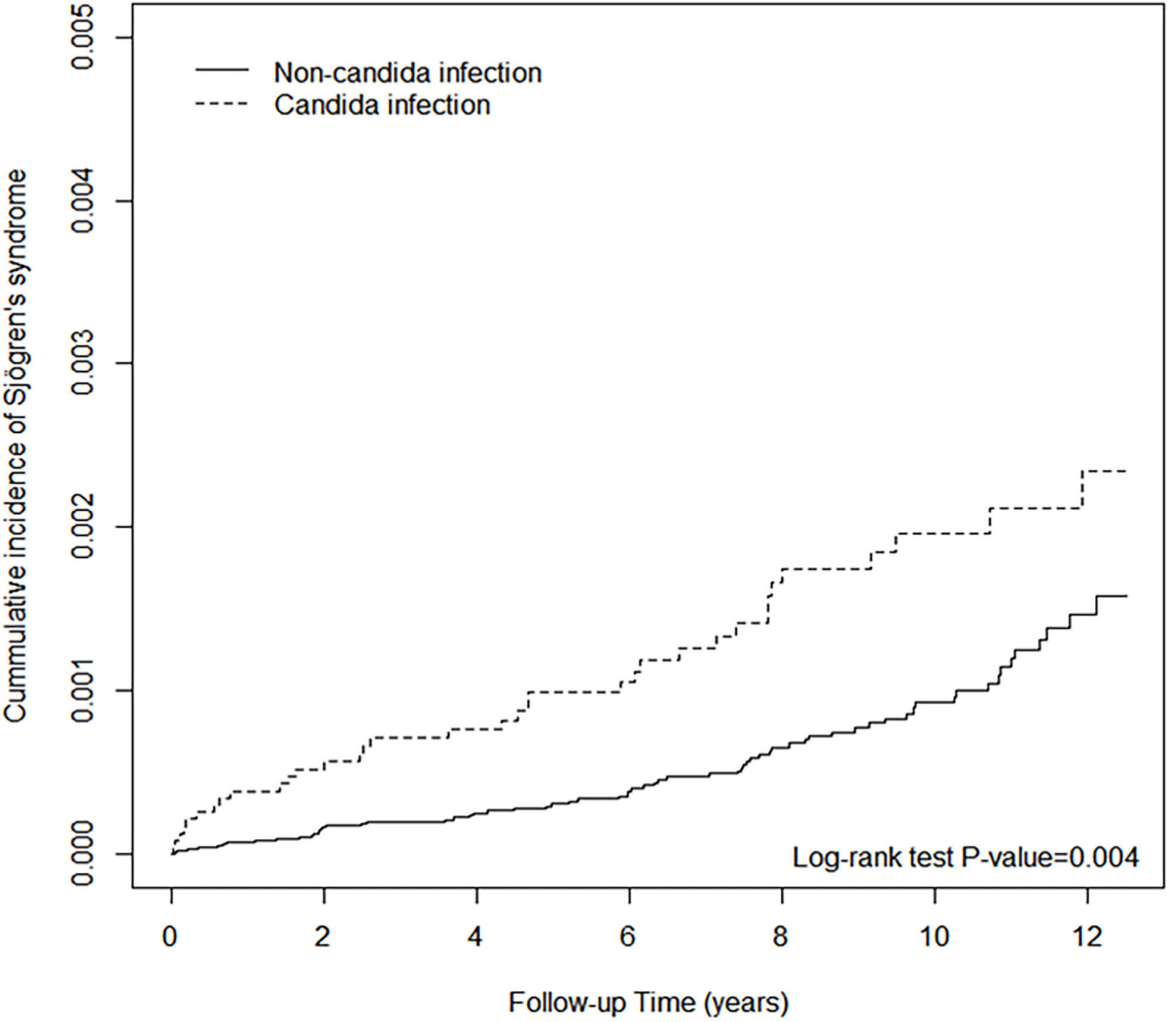
The cumulative incidence of Sjögren's syndrome of non-*Candida* infection and *Candida* infection.

Both univariable and multivariable Cox regression analyses showed that individuals with *Candida* infection had a higher risk of subsequent SjS compared with those without *Candida* infection, with an incidence rate of 1.92 per 10,000 person-years vs. 0.98 per 10,000 person-years (see Table 2). Individuals with a history of *Candida* infection were more likely to develop SjS (unadjusted HR, 1.83; 95 CI, 1.21–2.77). Figure 1 shows the survival curve. The cumulative incidence of SjS is higher in the *Candida* infection group than in the control group (*P* = 0.004). The results of multivariable Cox regression analysis are listed in Table 2, which reveals a positive correlation of *Candida* infection with the development of SjS. After adjusting for demographic variables and comorbidities at baseline, it was found that individuals with *Candida* infection had a 90% higher risk of developing subsequent SjS than those in the control group, with an adjusted HR of 1.90 (95% CI, 1.25–2.87). We also found that older participants were at a higher risk of developing SjS [adjusted HR (aHR) = 2.58, 95% CI, 1.38–4.80; aHR = 2.94, 95% CI, 1.51–5.74; aHR = 5.08, 95% CI, 2.58–9.97 in the age groups of 31–40, 41–50, and >50 years, respectively]. The participants with CLD, depression, allergic rhinitis, and urticaria also had a higher risk for subsequent SjS (with aHR = 1.84, 2.40, 1.79, 2.23, and 2.22, respectively; see Table 2). Patients with comorbidity and SLE had a significantly high relative risk of SjS (aHR = 11.1, 95% CI 4.38–28.4).

**Table 2:** The incidence rate and hazard ratio of Sjögren's syndrome.

	**Sjögren's syndrome**						
**Variables**	***n***	**PY**	**IR**	**cHR**	**(95% CI)**	**aHR^†^**	**(95% CI)**
Non-*Candida* infection	69	706,735	0.98	1.00	-	1.00	-
*Candida* infection	35	182,626	1.92	1.83	(1.21, 2.77)^**^	1.90	(1.25, 2.87)^**^
Sex							
Female	102	808,522	1.26	1.00	-	1.00	-
Male	2	80,838	0.25	0.20	(0.05, 0.83)^*^	0.14	(0.03, 0.58)^**^
Age, year							
18–30	16	373,712	0.43	1.00	-	1.00	-
31–40	27	228,671	1.18	2.65	(1.43, 4.92)^**^	2.58	(1.38, 4.80)^**^
41–50	20	134,520	1.49	3.34	(1.73, 6.44)^***^	2.94	(1.51, 5.74)^**^
>50	41	152,458	2.69	6.33	(3.55, 11.28)^***^	5.08	(2.58, 9.97)^***^
Occupation							
Officers	48	481,862	1.00	1.00	-	1.00	-
Worker	33	232,766	1.42	1.42	(0.91, 2.22)	1.24	(0.79, 1.94)
Fisher	0	131	0.00	0.00	(0,Inf)	0.00	(0,Inf)
Farmer	18	97,446	1.85	1.88	(1.1 ,3.24)^*^	1.51	(0.86, 2.66)
Other	5	77,156	0.65	0.66	(0.26, 1.65)	0.68	(0.27, 1.71)
Comorbidities							
Hypertension							
No	83	782,247	1.06	1.00	-	1.00	-
Yes	21	107,114	1.96	1.93	(1.19, 3.12)^**^	0.55	(0.30, 1.01)
Diabetes							
No	93	824,816	1.13	1.00	-		
Yes	11	64,544	1.70	1.59	(0.85, 2.97)		
Hyperlipidemia							
No	83	814,939	1.02	1.00	-	1.00	-
Yes	21	74,421	2.82	2.95	(1.83, 4.77)^***^	1.17	(0.65, 2.09)
CVA							
No	94	855,232	1.10	1.00	-	1.00	-
Yes	10	34,129	2.93	2.87	(1.49, 5.52)^**^	1.24	(0.59, 2.60)
CKD							
No	104	883,765	1.18	1.00	-		
Yes	0	5,596	0.00	0.00	(0,Inf)		
COPD							
No	87	834,453	1.04	1.00	-	1.00	-
Yes	17	54,908	3.10	3.19	(1.89, 5.37)^***^	1.56	(0.87,2.79)
CLD							
No	83	819,228	1.01	1.00	-	1.00	-
Yes	21	70,132	2.99	3.14	(1.95, 5.08)^***^	1.84	(1.09, 3.10)^*^
Depression							
No	90	853,540	1.05	1.00	-	1.00	-
Yes	14	35,821	3.91	4.03	(2.29, 7.1)^***^	2.40	(1.33 4.33)^**^
Allergic rhinitis							
No	74	732,008	1.01	1.00	-	1.00	-
Yes	30	157,353	1.91	2.13	(1.38, 3.27)^***^	1.79	(1.15, 2.79)^*^
Urticaria							
No	72	756,530	0.95	1.00	-	1.00	-
Yes	32	132,830	2.41	2.90	(1.90, 4.43)^***^	2.23	(1.45, 3.42)^***^
SLE							
No	99	886,707	1.12	1.00	-	1.00	-
Yes	5	2653	18.84	17.84	(7.26, 43.9)^***^	11.1	(4.38, 28.4)^***^
Rheumatic arthritis							
No	96	878,018	1.09	1.00	-	1.00	-
Yes	8	11,342	7.05	6.78	(3.3, 14.0)^***^	2.22	(1.02, 4.79)^*^

* *p < 0.05*;

** *p < 0.01*;

*** *p < 0.001*.

*CVA, cerebrovascular accident; CKD, chronic kidney disease; COPD, chronic obstructive pulmonary disease; CLD, chronic liver diseases; SLE, systemic lupus erythematosus PY, person-year; IR, incidence rate per 10,000 person-year; cHR, crude hazard ratio; aHR, adjusted hazard ratio*.

† *Adjusted by gender, age, occupation, hypertension, hyperlipidemia, CVA, COPD, CLD, depression, allergic rhinitis, urticaria, SLE and rheumatic arthritis*.

### Sensitivity Analyses

Supplementary Tables 1, 2 list a scenario to examine SjS events using the robustness of HR. We excluded patients with both SjS and SLE and RA during the follow up period (from the index date till the end of the study) to minimize the effect of secondary SjS and possible bias of collinearity. Sensitivity analysis showed the aHR = 1.95 (95% CI 1.14–3.35) for SjS with exposure to *Candida* infection.

Supplementary Tables 3, 4 list another scenario using case and control analysis. We identified 810 cases and 3,240 matched controls. *Candida* infection was associated with a 1.34-fold increase in the risk of SjS (adjusted OR (aOR) 1.34, 95% CI 1.03–1.73, *P* < 0.05).

### Subgroup Analysis

Table 3 lists the results of different stratifications. The analysis of the sex subgroup showed that women with *Candida* infection have a higher risk of developing SjS (aHR = 1.87, 95% CI 1.23–2.85; *P* < 0.01), while men with *Candida* infection did not show any significant association (aHR = 4.08, 95% CI 0.25–66.33). The analysis of the age subgroup revealed that, compared with the age-matched non-*Candida* subgroup, 18–30-year-old participants with *Candida* infection had a significantly higher risk of developing SjS (aHR = 4.30, 95% CI 1.60–11.55; *P* < 0.01). The analysis of the comorbidity subgroup showed that, in patients with depression, the presence of *Candida* infection is associated with a significantly increased risk of the occurrence of SjS than in those not exposed to *Candida* infection (aHR = 6.34, 95% CI 2.16–18.66; *P* < 0.001). Table 4 shows the risk of SjS associated with the occurrence of *Candida* infection at different sites. Patients with oral *Candida* infection are associated with the subsequent diagnosis of SjS (aHR = 4.70, 95% CI 4.70–13.39; *P* < 0.01). Patients with *Candida* infection at the vulva/vagina and skin/nails are also associated with the subsequent SjS (aHR = 1.56, 95% CI 1.56–2.53 and aHR = 1.92, 95% CI 1.92–9.40, respectively).

**Table 3:** The risk of Sjögren's syndrome in different stratification.

	**Non-*****Candida*** **infection**			***Candida*** **infection**						
**Variables**	***n***	**PY**	**IR**	***n***	**PY**	**IR**	**cHR**	**(95% CI)**	**aHR^†^**	**(95% CI)**
Sex										
Female	68	641,711	1.06	34	166,811	2.04	1.79	(1.18, 2.73)^**^	1.87	(1.23, 2.85)^**^
Male	1	65,024	0.15	1	15,814	0.63	4.18	(0.26, 66.8)	4.08	(0.25, 66.33)
Age, year										
18–30	8	301,235	0.27	8	72,477	1.10	4.08	(1.53, 10.88)^**^	4.30	(1.6, 11.55)^**^
31–40	20	178,563	1.12	7	50,107	1.40	1.22	(0.52, 2.9)	1.20	(0.51, 2.85)
41–50	11	103,739	1.06	9	30,781	2.92	2.74	(1.13, 6.63)^*^	2.69	(1.11, 6.5)^*^
>50	30	123,197	2.44	11	29,261	3.76	1.29	(0.63, 2.66)	1.44	(0.7, 2.98)
Occupation										
Officers	34	383,463	0.89	14	98,399	1.42	1.47	(0.79, 2.76)	1.48	(0.79, 2.78)
Worker	20	183,000	1.09	13	49,766	2.61	2.27	(1.11, 4.65)^*^	2.34	(1.14, 4.81)^*^
Fisher	0	107	0.00	0	24	0.00				
Farmer	11	78,665	1.40	7	18,781	3.73	2.50	(0.96, 6.51)	3.03	(1.14, 8.07)^*^
Other	4	61,501	0.65	1	15,655	0.64	0.99	(0.11, 8.87)	1.20	(0.13, 10.73)
Comorbidities										
Hypertension										
No	54	619,455	0.87	29	162,791	1.78	1.90	(1.2, 3.01)^**^	1.92	(1.21, 3.04)^**^
Yes	15	87,279	1.72	6	19,835	3.03	1.63	(0.62, 4.23)	1.88	(0.72, 4.93)
Diabetes										
No	60	654,487	0.92	33	170,329	1.94	1.98	(1.28 ,3.04)^**^	2.01	(1.3, 3.1)^**^
Yes	9	52248	1.72	2	12,296	1.63	0.90	(0.19, 4.15)	0.95	(0.2, 4.45)
Hyperlipidemia										
No	55	647,183	0.85	28	167,756	1.67	1.80	(1.13, 2.87)^*^	1.84	(1.16,2.94)^*^
Yes	14	59,552	2.35	7	14,869	4.71	1.97	(0.8, 4.89)	2.08	(0.84, 5.15)
CVA										
No	61	678,330	0.90	33	176,902	1.87	1.93	(1.25, 2.96)^**^	1.94	(1.26, 2.99)^**^
Yes	8	28,404	2.82	2	5,724	3.49	1.23	(0.26, 5.81)	1.42	(0.3, 6.79)
CKD										
No	69	702,108	0.98	35	181,657	1.93	1.83	(1.21, 2.77)^**^	1.90	(1.25, 2.87)^**^
Yes	0	4,627	0.00	0	969	0.00				
COPD										
No	56	661,140	0.85	31	173,313	1.79	2.05	(1.32, 3.18)^**^	2.03	(1.3, 3.16)^**^
Yes	13	45,595	2.85	4	9,313	4.30	1.04	(0.3, 3.61)	1.24	(0.36, 4.27)
CLD										
No	56	650,564	0.86	27	168,664	1.60	1.72	(1.08, 2.74)^*^	1.76	(1.1, 2.81)^*^
Yes	13	56,171	2.31	8	13,961	5.73	2.36	(0.98, 5.71)	2.67	(1.09, 6.55)^*^
Depression										
No	63	677,872	0.93	27	175,668	1.54	1.51	(0.95, 2.39)	1.53	(0.97, 2.43)
Yes	6	28,863	2.08	8	6,958	11.50	5.58	(1.93, 16.07)^**^	6.34	(2.16, 18.66)^***^
Allergic rhinitis										
No	49	579,887	0.84	25	152,120	1.64	1.77	(1.08, 2.89)^*^	1.79	(1.09, 2.93)^*^
Yes	20	126,847	1.58	10	30,505	3.28	2.04	(0.95, 4.36)	2.30	(1.07, 4.95)^*^
Urticaria										
No	47	599,691	0.78	25	156,840	1.59	1.83	(1.11, 3)^*^	1.83	(1.11, 3.01)^*^
Yes	22	107,044	2.06	10	25,786	3.88	1.90	(0.9, 4.02)	2.12	(1, 4.52)
SLE										
No	64	704,610	0.91	35	182,097	1.92	1.97	(1.3, 2.99)^**^	2.05	(1.35, 3.12)^***^
Yes	5	2,124	23.54	0	529	0.00	0.00	(0,Inf)	0.00	(0,Inf)
Rheumatic arthritis										
No	61	697,791	0.87	35	180,227	1.94	2.06	(1.35, 3.15)^***^	2.16	(1.41, 3.3)^***^
Yes	8	8,943	8.95	0	2,399	0.00	0.00	(0, Inf)	0.00	(0, Inf)

* *p < 0.05*;

** *p < 0.01*;

*** *p < 0.001*.

*PY, person-year; IR, incidence rate per 10,000 person-year; cHR, crude hazard ratio; aHR, adjusted hazard ratio*.

† *Adjusted by gender, age, hypertension, hyperlipidemia, CVA, COPD, CLD, depression, allergic rhinitis, urticaria, SLE and rheumatic arthritis*.

**Table 4:** The risk of Sjögren's syndrome in different sites of *Candida* infection.

	**Sjögren's syndrome**						
**Variables**	***n***	**PY**	**IR**	**cHR**	**(95% CI)**	**aHR^†^**	**(95% CI)**
*Candida* infection-Mouth							
No	69	706,735	0.98	1.00	-	1.00	-
Yes	4	5,401	7.41	6.25	(2.1, 18.58)^***^	4.70	(4.7, 13.93)^**^
*Candida* infection-Vulva and vagina							
No	69	706,735	0.98	1.00	-	1.00	-
Yes	24	148,028	1.62	1.49	(0.93 ,2.4)	1.56	(1.56, 2.53)
*Candida* infection-Skin and nails							
No	69	706,735	0.98	1.00	-	1.00	-
Yes	2	5,987	3.34	2.34	(0.49, 11.22)	1.92	(1.92, 9.40)

** *p < 0.01*;

*** *p < 0.001*.

*PY, person-year; IR, incidence rate per 10,000 person-year; cHR, crude hazard ratio; aHR, adjusted hazard ratio*.

† *Adjusted by gender, age, hypertension, hyperlipidemia, CVA, COPD, CLD, depression, allergic rhinitis, urticaria, SLE and rheumatic arthriti*.

## Discussion

To the best of our knowledge, this is the first retrospective observation study that used nation-wide population-based data to investigate the link between *Candida* infection and the risk of SjS. The results showed that patients with *Candida* infection have a 1.90-fold higher risk of developing SjS compared to the controls; those with oral *Candida* infection have a 4.70-fold increased risk of SjS. Furthermore, the positive association has been consistently observed in sensitivity analysis models.

Previous studies have reported an incidence rate of 3–11 per 100 000 person-years for SjS (2–5). Our study showed that women have a higher incidence rate of SjS (12.6 per 100,000 person-years) than men; similar results have been reported in a previous study too (17). Our study also showed that the participants with comorbidity and SLE have a significantly higher relative risk of SjS. It has been reported that women, particularly those in their menopause, comprise a greater proportion of patients with SjS (18) and that oral *Candida* colonization and infection is a common clinical manifestation in patients with SjS (19, 20). A previous study has shown that the sicca syndrome, fatigue, and non-specific musculoskeletal pain in patients with SjS may be mistaken for aging, and that depression in patients with pSS and systemic manifestations of SjS can sometimes occur prior to the sicca syndrome (21). In the present study, we showed that patients diagnosed with depression at baseline with a *Candida* infection were at a significantly higher risk of being diagnosed with SjS later. Our study results are consistent with those of previous studies. In addition, our study provides more information on the long-term cumulative incidence and role of *Candida* infection before the diagnosis of SjS.

The potential role of and the possible mechanism through which *Candida* infection triggers or amplifies the pathway to SjS is still unknown. Multiple factors are responsible for the occurrence of SjS. Using animal models, some studies have suggested that the *Candida* infection is an important environmental factor responsible for SjS (22). Mofors et al. reported an increased likelihood of developing SjS following a history of infections (23). *Candida* spp. are common commensal fungi, some of which can act as pathogens under certain conditions. Therefore, it is necessary to carefully investigate the relation between the *Candida* infection and SjS. *Candida* induces the human Th17 cell response, which plays an important physiological role in the development of SjS (24, 25). SjS patients have been shown to have significantly higher serum levels of galectin-3, which plays a possibly active role in host defense against *Candida* infection (26–29). The pivotal role of galectin-3 in autoimmune diseases and neutrophil phagocytosis of *Candida* infection (28, 30) implies that *Candida* infection and SjS share a common pathological pathway. Thirdly, candidalysin, a toxin secreted by *Candida albicans*, can damage the oral epithelial membrane and trigger a double-sided blade signal pathway that activates epithelial immunity (31). Interleukin-17 signaling is crucial for both innate and adaptive immunity to *C*. *albicans*. Interleukin-17 expression increases in the patients with SjS. The interplay between candidalysin and interleukin-17 sets a self-reinforcing loop, leading to inflammation amplification (32).

Our study has several limitations as well. First, there are at least 15 species of *Candida* that can infect humans, and *Candida albicans* is the most prevalent one. However, the NHIRD could not provide reports on microbiological cultures, and therefore, we could not identify the *Candida* species in detail, which prevents us from performing a more comprehensive analysis to explore the relationship between *C. albicans* and non-*C. albicans* infection and the occurrence of SjS. Second, this database cannot provide any information about the inflammatory marker either; in addition, the diagnosis of SS should not be based solely on SSA/Ro and/or SSB/La antibodies, as these antibodies can be found in healthy people too. Third, the diagnosis of *Candida* infection and SjS is based on the ICD-9-CM coded data and not on prospective clinical collections. A misclassification bias may also occur. The diagnosis of *Candida* infection depends primarily on the clinical appearance and/or the results of specimen examinations. Some patients with mild clinical symptoms of *Candida* infection did not seek medical advice, and hence, they may have been included in the control group. Such situations may lead to type 2 errors. However, if the *Candida* infection is supposed to be positively associated with the subsequent SjS, such misclassification would bias the estimated HRs toward the null. Therefore, more research should be performed by including participants from other countries and ethnic cultures as well to confirm and extrapolate our findings.

## Conclusion

This study suggests that *Candida* infection is associated with a higher incidence of SjS thereafter, particularly in individuals with oral *Candida* infection, or concomitant depression. We suggested that, in some cases, *Candida* infection should be considered along with other criteria for the early diagnosis of SjS.

## Data Availability Statement

The original contributions presented in the study are included in the article/Supplementary Material, further inquiries can be directed to the corresponding author/s.

## Ethics Statement

This study was approved by the Institutional Review Board of China Medical University Hospital Research Ethics Committee (CMUH104-REC2-115(AR-4). Written informed consent for participation was not required for this study in accordance with the national legislation and the institutional requirements.

## Author Contributions

C-LC, M-CC, RC, and JC-CW: conceptualization. H-TY: data curation and funding acquisition. C-LC, F-CC, Y-MH, M-CC, RC, and JC-CW: formal analysis and methodology. C-LC and F-CC: investigation. C-LC and Y-MH: writing (original draft preparation). RC, JC-CW, and M-CC: writing (review and editing). All authors contributed to the article and approved the submitted version.

## Conflict of Interest

The authors declare that the research was conducted in the absence of any commercial or financial relationships that could be construed as a potential conflict of interest.

## Publisher's Note

All claims expressed in this article are solely those of the authors and do not necessarily represent those of their affiliated organizations, or those of the publisher, the editors and the reviewers. Any product that may be evaluated in this article, or claim that may be made by its manufacturer, is not guaranteed or endorsed by the publisher.
