# Supplementary material for: Candida Infection as an Early Sign of Subsequent Sjögren's Syndrome: A Population-Based Matched Cohort Study
Source: Front Med (Lausanne). 2022 Jan 21;8:796324. doi: 10.3389/fmed.2021.796324 (PMC8814339; doi:10.3389/fmed.2021.796324)
Supplement: Supplementary file 1 [file Data_Sheet_1.docx]

Supplement Table 1. Demographics in sensitivity analysis (I)

|  | non-*Candida* infection | |  | *Candida* infection | |  |
| --- | --- | --- | --- | --- | --- | --- |
|  | N= 92 592 | |  | N=23 148 | |  |
| Variables | n | % |  | n | % | SMD |
| Sex |  |  |  |  |  | 0.001 |
| Female | 82864 | 89% |  | 20170 | 87% |  |
| Male | 9728 | 11% |  | 2438 | 11% |  |
| Age, year |  |  |  |  |  |  |
| 18-30 | 39810 | 43% |  | 9821 | 42% | 0.01 |
| 31-40 | 22544 | 24% |  | 5830 | 25% | 0.02 |
| 41-50 | 13657 | 15% |  | 3332 | 14% | 0.01 |
| >50 | 16581 | 18% |  | 4165 | 18% | 0.002 |
| mean, (SD) | 36.7 | (15.3) |  | 37.1 | (15.8) | 0.03 |
| Occupation |  |  |  |  |  |  |
| officers | 50114 | 54% |  | 12322 | 53% | 0.02 |
| worker | 24066 | 26% |  | 6220 | 27% | 0.02 |
| fisher | 7 | 0.01% |  | 3 | 0.01% | 0.01 |
| farmer | 10349 | 11% |  | 2552 | 11% | 0.01 |
| other | 8056 | 8.7% |  | 2051 | 8.9% | 0.01 |
| Comorbidities |  |  |  |  |  |  |
| hypertension | 11673 | 13% |  | 2984 | 13% | 0.01 |
| diabetes | 7186 | 7.8% |  | 1880 | 8.1% | 0.01 |
| hyperlipidemia | 8364 | 9.0% |  | 2161 | 9.3% | 0.01 |
| CVA | 4122 | 4.5% |  | 1131 | 4.9% | 0.01 |
| CKD | 760 | 0.8% |  | 218 | 0.9% | 0.01 |
| COPD | 6112 | 6.6% |  | 1603 | 6.9% | 0.01 |
| CLD | 7517 | 8.1% |  | 1925 | 8.3% | 0.01 |
| depression | 3794 | 4.1% |  | 1085 | 4.7% | 0.03 |
| allergic rhinitis | 18595 | 20% |  | 4630 | 20% | 0.002 |
| urticaria | 16017 | 17% |  | 4031 | 17% | 0.003 |

SMD: standard mean difference;
CVA: cerebrovascular accident; CKD: chronic kidney disease; COPD: chronic obstructive pulmonary disease; CLD: chronic liver diseases.

Supplemnt Table 2. Risk factors of Sjögren's syndrome in sensitivity analysis (I)

|  | Sjögren's syndrome | | |  |  |  |  |
| --- | --- | --- | --- | --- | --- | --- | --- |
| Variables | n | PY | IR | cHR | (95% CI) | aHR | (95% CI) |
| non-*Candida* infection | 40 | 720045 | 0.56 | 1.00 | - | 1.00 | - |
| *Candida* infection | 20 | 182762 | 1.09 | 1.92 | (1.12,3.29)* | 1.95 | (1.14,3.35)* |
| Gender |  |  |  |  |  |  |  |
| Female | 56 | 820573 | 0.68 | 1.00 | - |  |  |
| Male | 4 | 82233 | 0.49 | 0.74 | (0.27,2.05) |  |  |
| Age, year |  |  |  |  |  |  |  |
| 18-30 | 11 | 383291 | 0.29 | 1.00 | - | 1.00 | - |
| 31-40 | 9 | 230977 | 0.39 | 1.30 | (0.54,3.15) | 1.20 | (0.5,2.91) |
| 41-50 | 21 | 144864 | 1.45 | 4.77 | (2.3,9.91)*** | 3.90 | (1.85,8.21)*** |
| >50 | 19 | 143675 | 1.32 | 4.70 | (2.24,9.89)*** | 2.97 | (1.24,7.11)* |
| Occupation |  |  |  |  |  |  |  |
| officers | 26 | 493129 | 0.53 | 1.00 | - | 1.00 | - |
| worker | 19 | 238290 | 0.80 | 1.50 | (0.83,2.72) | 1.26 | (0.69,2.28) |
| fisher | 0 | 68 | 0 |  |  |  |  |
| farmer | 10 | 94738 | 0.53 | 2.08 | (1,4.31)* | 1.67 | (0.79,3.54) |
| other | 5 | 76581 | 0.65 | 1.26 | (0.48,3.28) | 1.22 | (0.47,3.2) |
| Comorbidities |  |  |  |  |  |  |  |
| hypertension |  |  |  |  |  |  |  |
| No | 48 | 806881 | 0.59 | 1.00 | - | 1.00 | - |
| Yes | 12 | 95925 | 1.25 | 2.29 | (1.21,4.32)* | 0.78 | (0.36,1.71) |
| diabetes |  |  |  |  |  |  |  |
| No | 54 | 845141 | 0.64 | 1.00 | - |  |  |
| Yes | 6 | 57665 | 1.04 | 1.77 | (0.76,4.13) |  |  |
| hyperlipidemia |  |  |  |  |  |  |  |
| No | 50 | 835614 | 0.60 | 1.00 | - | 1.00 | - |
| Yes | 10 | 67192 | 1.49 | 2.79 | (1.41,5.53)** | 0.96 | (0.43,2.15) |
| CVA |  |  |  |  |  |  |  |
| No | 56 | 873759 | 0.64 | 1.00 | - |  |  |
| Yes | 4 | 29047 | 1.38 | 2.39 | (0.86,6.62) |  |  |
| CKD |  |  |  |  |  |  |  |
| No | 60 | 898426 | 0.67 | 1.00 | - |  |  |
| Yes | 0 | 4380 | 0.00 |  |  |  |  |
| COPD |  |  |  |  |  |  |  |
| No | 53 | 856714 | 0.62 | 1.00 | - | 1.00 | - |
| Yes | 7 | 46093 | 1.52 | 2.81 | (1.27,6.23)* | 1.33 | (0.56,3.13) |
| CLD |  |  |  |  |  |  |  |
| No | 46 | 840273 | 0.55 | 1.00 | - | 1.00 | - |
| Yes | 14 | 62533 | 2.24 | 4.70 | (2.57,8.61)*** | 2.77 | (1.43,5.36)** |
| depression |  |  |  |  |  |  |  |
| No | 49 | 873460 | 0.56 | 1.00 | - | 1.00 | - |
| Yes | 11 | 29346 | 3.75 | 7.67 | (3.96,14.84)*** | 4.82 | (2.41,9.61)*** |
| allergic rhinitis |  |  |  |  |  |  |  |
| No | 47 | 762793 | 0.62 | 1.00 | - |  |  |
| Yes | 13 | 140014 | 0.93 | 1.83 | (0.97,3.42) |  |  |
| urticaria |  |  |  |  |  |  |  |
| No | 43 | 781658 | 0.55 | 1.00 | - | 1.00 | - |
| Yes | 17 | 121148 | 1.40 | 3.16 | (1.78,5.63)*** | 2.58 | (1.44,4.6)** |

*: p-value<0.05; **: p-value<0.01; ***: p-value<0.001;
PY: person-years; IR: incidence rate per 10000 person-year; cHR: crude hazard ratio; aHR: adjusted hazard ratio;
†: adjusted by gender, age, occupation, hypertension, hyperlipidemia, COPD, CLD, depression and urticaria.

Supplement Table 3. Demographics in sensitivity analysis (II)

| Case-control Study | | | | | | |
| --- | --- | --- | --- | --- | --- | --- |
|  | non-Sjögren's syndrome | |  | Sjögren's syndrome | |  |
|  | N=3240 | |  | N=810 | |  |
| Variables | n | % |  | n | % | p-value |
| Sex |  |  |  |  |  | 1.00 |
| Female | 2844 | 87% |  | 711 | 88% |  |
| Male | 396 | 13% |  | 99 | 12% |  |
| Age, year |  |  |  |  |  | 1.00 |
| 18-30 | 157 | 5% |  | 40 | 5% |  |
| 31-40 | 412 | 13% |  | 103 | 13% |  |
| 41-50 | 632 | 20% |  | 158 | 20% |  |
| >50 | 2039 | 63% |  | 509 | 63% |  |
| mean, (SD) | 54.2 | (14.4) |  | 54.2 | (14.2) | 1.00 |
| Occupation |  |  |  |  |  | 0.99 |
| officers | 1828 | 56% |  | 457 | 56% |  |
| worker | 808 | 25% |  | 205 | 25% |  |
| fisher | 0 | 0% |  | 0 | 0% |  |
| farmer | 368 | 11% |  | 89 | 11% |  |
| other | 236 | 7% |  | 59 | 7% |  |
| Comorbidities |  |  |  |  |  |  |
| hypertension | 1104 | 34% |  | 267 | 33% | 0.55 |
| diabetes | 560 | 17% |  | 133 | 16% | 0.56 |
| hyperlipidemia | 905 | 28% |  | 263 | 32% | 0.01 |
| CVA | 315 | 10% |  | 119 | 15% | <0.001 |
| CKD | 58 | 2% |  | 24 | 3% | 0.03 |
| COPD | 443 | 14% |  | 186 | 23% | <0.001 |
| CLD | 480 | 15% |  | 252 | 31% | <0.001 |
| depression | 263 | 8% |  | 154 | 19% | <0.001 |
| allergic rhinitis | 775 | 24% |  | 351 | 43% | <0.001 |
| urticaria | 682 | 21% |  | 294 | 36% | <0.001 |
| SLE | 18 | 1% |  | 122 | 15% | <0.001 |
| rheumatic arthritis | 130 | 4% |  | 197 | 24% | <0.001 |

CVA: cerebrovascular accident; CKD: chronic kidney disease; COPD: chronic obstructive pulmonary disease; CLD: chronic liver diseases; SLE: systemic lupus erythematosus

Supplement Table 4. The odds ratio of *Candida* Infection and Sjögren's syndrome

|  | Candidiasis Infection | | |  |  |  |  |
| --- | --- | --- | --- | --- | --- | --- | --- |
|  | n | N | ratio | cOR | (95% CI) | aOR | (95% CI) |
| Control | 277 | 3240 | 0.09 | 1.00 | (Reference) | 1.00 | (Reference) |
| Case | 100 | 810 | 0.12 | 1.51 | (1.18,1.92)*** | 1.34 | (1.03,1.73)* |
|  |  |  |  |  |  |  |  |

*: p-value<0.05; **: p-value<0.01; ***: p-value<0.001;

Control: non-Sjögren's syndrome; Case: Sjögren's syndrome
cOR: crude odds ratio; aOR: adjusted odds ratio;
†: adjusted by sex, age, occupation and all comorbiditie
